# Self-Healing Dynamic Hydrogel Microparticles with Structural Color for Wound Management

**DOI:** 10.1007/s40820-024-01422-4

**Published:** 2024-07-02

**Authors:** Li Wang, Xiaoya Ding, Lu Fan, Anne M. Filppula, Qinyu Li, Hongbo Zhang, Yuanjin Zhao, Luoran Shang

**Affiliations:** 1 Department of General Surgery, Ruijin Hospital, Shanghai Jiaotong University School of Medicine, Shanghai, 200025 People’s Republic of China; 2 Department of Rheumatology and Immunology, Nanjing Drum Tower Hospital, School of Biological Science and Medical Engineering, Southeast University, Nanjing, 210096 People’s Republic of China; 3 Pharmaceutical Sciences Laboratory, Åbo Akademi University, 20520 Turku, Finland; 4 Oujiang Laboratory (Zhejiang Lab for Regenerative Medicine, Vision and Brain Health), Wenzhou Institute, University of Chinese Academy of Sciences, Wenzhou, 325001 People’s Republic of China; 5 Shanghai Xuhui Central Hospital, Zhongshan-Xuhui Hospital, and the Shanghai Key Laboratory of Medical Epigenetics, the International Co-laboratory of Medical Epigenetics and Metabolism (Ministry of Science and Technology), Institutes of Biomedical Sciences, Fudan University, Shanghai, 200032 People’s Republic of China

**Keywords:** Black phosphorus, Structural color, Dynamic hydrogel, Inverse opal, Wound management

## Abstract

Chronic diabetic wounds confront a significant medical challenge because of increasing prevalence and difficult-healing circumstances. It is vital to develop multifunctional hydrogel dressings, with well-designed morphology and structure to enhance flexibility and effectiveness in wound management. To achieve these, we propose a self-healing hydrogel dressing based on structural color microspheres for wound management. The microsphere comprised a photothermal-responsive inverse opal framework, which was constructed by hyaluronic acid methacryloyl, silk fibroin methacryloyl and black phosphorus quantum dots (BPQDs), and was further re-filled with a dynamic hydrogel. The dynamic hydrogel filler was formed by Knoevenagel condensation reaction between cyanoacetate and benzaldehyde-functionalized dextran (DEX-CA and DEX-BA). Notably, the composite microspheres can be applied arbitrarily, and they can adhere together upon near-infrared irradiation by leveraging the BPQDs-mediated photothermal effect and the thermoreversible stiffness change of dynamic hydrogel. Additionally, eumenitin and vascular endothelial growth factor were co-loaded in the microspheres and their release behavior can be regulated by the same mechanism. Moreover, effective monitoring of the drug release process can be achieved through visual color variations. The microsphere system has demonstrated desired capabilities of controllable drug release and efficient wound management. These characteristics suggest broad prospects for the proposed composite microspheres in clinical applications.

**Supplementary Information:**

The online version contains supplementary material available at 10.1007/s40820-024-01422-4.

## Highlights

- Derived from silica photonic crystals, inverse opal microspheres have a regularly connected porous structure and inherit structural color properties.
- Combined with the stable scaffold and the photothermal phase-transition of the secondary filling material, the inverse opal composite microspheres are endowed with self-healing properties and the ability for controllable drug release.
- Inverse opal microspheres were significantly treated for diabetic wound, via promoting tissue regeneration, collagen deposition and angiogenesis. Meanwhile, the release of drugs could be monitored by the structural color characteristic.

**Supplementary Information:**

The online version contains supplementary material available at 10.1007/s40820-024-01422-4.

## Graphical abstract

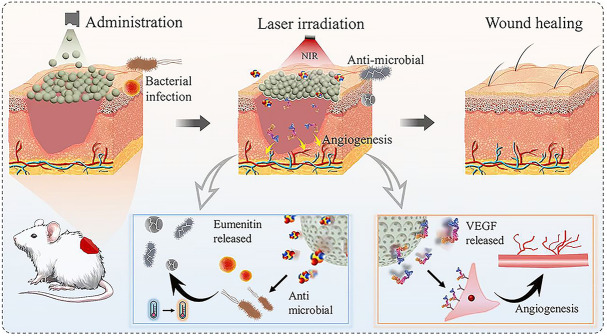

## Introduction

Chronic wounds, characterized by difficult healing and persistent inflammation, impose substantial economic and psychophysical burdens on patients [1, 2]. Various treatments have been developed for wound management, such as surgical debridement, negative pressure therapy, and the use of various dressings [3–6]. Among these, hydrogel dressings are considered effective taking advantage of their biocompatibility and the water-rich environment [7–13]. To enhance the multifunctionality of wound dressings, innovative structural designs have been incorporated into hydrogel systems [14–17]. A representative example is inverse opal hydrogel patches, whose periodic porous architecture gives rise to structural colors [18–20]. Inverse opal-based patches can not only carry various therapeutic agents, such as antimicrobial peptides (AMPs) and vascular endothelial growth factor (VEGF), but also utilize their structural color features for reporting the drug release progress [21–23]. Such multifunctionality makes inverse opal-based structural color patches highly favorable for smart wound management [24]. Despite these great advancements, bulk structural color films face dilemmas of inferior flexibility and breathability, making it difficult to adjust to diverse application scenarios. Additionally, the angular variability of the reflected color may lead to inaccuracies in sensing [25]. Therefore, further development of novel smart structural color patches addressing these challenges is needed.

**Fig. 1:**
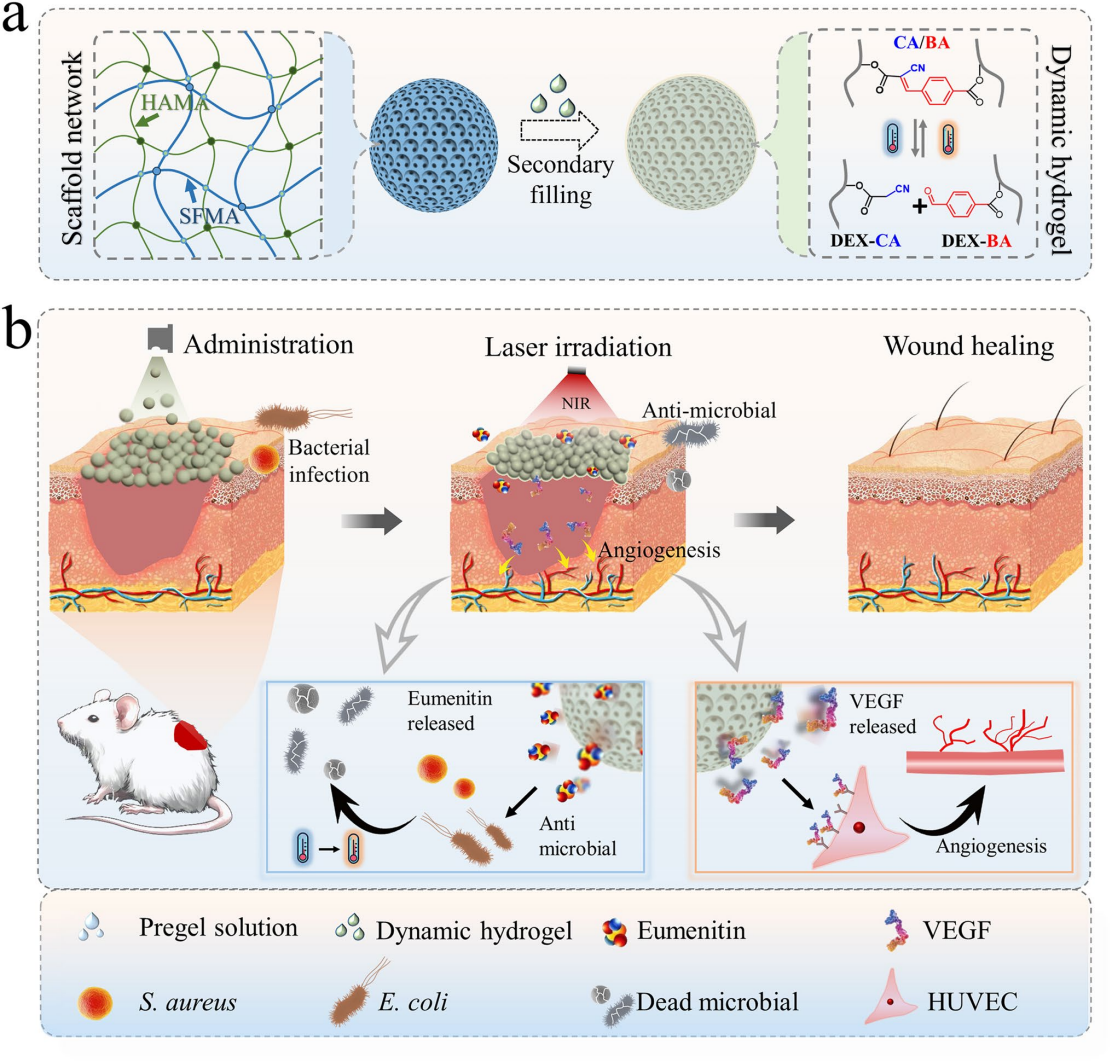
Diagram of the intelligent responsive structural color CMPs for wound management. **a** Fabrication of the inverse opal microspheres compounded with a dynamic hydrogel as the filler. **b** Application of the CMPs to wounds, which can cover irregular wound area, fight against bacteria, and promote angiogenesis

In this paper, we propose black phosphorus-doped inverse opal microspheres (IOMPs) with shape reconfigurability and precise sensing capabilities for wound management, as schemed in Fig. 1. Inverse opals are known to possess a periodic interconnected porous structure obtained through replication of closely packed nanoparticle assemblies [26, 27]. This distinctive feature imparts them with specific photonic bandgap effects, and different active ingredients can be incorporated in either the inverse opal scaffold or secondary filling materials [28, 29]. In contrast to inverse opal films, IOMPs show angle-independent structural color, holding more reliable values for sensing purposes [25, 30]. Another important factor when considering the requirement for wound management is the control over the shape adaptability of the dressings [31–33]. One effective strategy is to impart microspheres with stimuli-responsive self-healing ability so that they can aggregate to form an impact scaffold for stable drug release, while maintaining a higher extent of flexibility and breathability compared to bulk films [34, 35]. Thus, numerous efforts have been devoted to the development of such dressings. Among the fascinating candidates, hydrogels synthesized via Knoevenagel condensation (KC) reaction based on benzaldehyde and cyanoacetate have attracted much attention [36–38]. The formation of reversible dynamic bond in the KC reaction, which could be catalyzed by histidine, enables the hydrogel to exhibit tunable stiffness and functionality [39]. Thus, the combination of self-healing hydrogel and IOMPs offers a possible solution to realize intelligent management of wounds.

Herein, we constructed composite IOMPs with desirable properties by combining a photothermal-responsive inverse opal framework with a dynamic hydrogel as the secondary filling material. The inverse opal framework was composed of hyaluronic acid methacryloyl (HAMA) and silk fibroin methacryloyl (SFMA) doped with black phosphorus quantum dots (BPQDs), and the dynamic hydrogel filler was formed by KC reaction between cyanoacetate and benzaldehyde-functionalized dextran (DEX-CA and DEX-BA). Notably, composite hydrogel microspheres could be applied arbitrarily by spraying, and they were able to adhere together upon near-infrared (NIR) irradiation by leveraging the BPQDs-mediated photothermal effect and the thermoreversible stiffness change of the dynamic hydrogel. Additionally, AMP and VEGF were co-loaded in the microspheres and their release behavior was regulated by the same mechanism. Moreover, effective monitoring of the drug release process was achieved through visual color variations. Based on these features, the composite microspheres (CMPs) demonstrated outstanding capabilities for wound repair in vivo. These results highlighted the valuable potential of the CMPs in biomedical applications.

## Experimental Section

### Materials

Dextran (Mn = 70 kDa), 1-ethyl-3-(3-dimethylaminopropyl) carbodiimide hydrochloride (EDC·HCl), and dimethyl sulfoxide (DMSO) were procured from Aladdin. 4-Formylbenzoic acid and cyanoacetic acid were obtained from Alfa Aesar. Black phosphorus quantum dots (BPQDs) were sourced from Nanjing XF NANO Technology Co., Ltd. Silica nanoparticles were synthesized using the Stöber method. Photonic crystal microspheres (PCMs), HAMA, SFMA, DEX-BA, and DEX-CA were synthesized based on our group’s prior works [31]. Additional reagents, such as methacrylic anhydride, were all procured from Sigma-Aldrich. Ultra-pure water utilized in the experiments was produced using a laboratory-grade water purification system (Millipore Milli-Q Plus 185).

### Synthesis of HAMA/SFMA Inverse Opal Microspheres

The silica photonic crystal microspheres (SPCMs) were fabricated by confined assembly of silica nanoparticles in microfluidic droplets. To achieve an inverse opal structure, we thoroughly impregnated dried SPCMs with a UV-polymerizable hydrogel precursor, primarily composed of 10% HAMA, 4% SFMA, 0.2 mg mL^−1^ BPQDs, and 1% photoinitiator HMPP. After approximately 4 h of incubation, the prepolymer filled the nano-channels of the SPCM template. Subsequently, a UV lamp was used to solidify the hydrogel, resulting in hybrid microspheres embedded within the hydrogel. Then, the hybrid microspheres were immersed in 4% hydrofluoric acid for 6 h to remove the silica nanoparticles, followed by multiple rinses with purified water to eliminate excess hydrofluoric acid.

### Fabrication and Characterization of Thermally Responsive DEX-BA/CA Hydrogel

We successfully synthesized DEX-BA and DEX-CA monomers and verified their characteristic peaks through nuclear magnetic resonance spectroscopy (QUANTUM-I-400 MHz). To construct DEX-CA/BA hydrogels, DEX-CA and DEX-BA with a molar ratio of cyanoacetate to benzaldehyde of 1:1 were dissolved in water. Additionally, 1% histidine was added to the system to catalyze the KC reaction. To investigate the effect of concentration on temperature sensitivity, bulk DEX-BA/CA hydrogels with varying concentrations (4%, 6%, 8%, and 10%) were incubated at 42 and 37 °C for 30 min, respectively. The changes in viscosity were recorded for each group. To prepare CMPs, IOMPs were initially dried at 37 °C for 2 h, followed by immersion in a pregel solution of DEX-CA/BA without histidine. Once the pregel solution thoroughly infiltrated the pores of IOMPs, histidine was added to induce rapid solidification of the pregel solution. To prepare CMPs, IOMPs were initially dried at 37 °C for 2 h, followed by immersion in a pregel solution of DEX-CA/BA without histidine. It is worth noting that the drugs were first dissolved in DEX-CA solution and then mixed with DEX-BA. Centrifugation or vacuum was used to promote dynamic hydrogel filling. Once the pregel solution thoroughly infiltrated the pores of IOMPs, histidine was added to induce rapid solidification of the pregel solution. The resulting bulk was immersed in PBS buffer and gently stirred to obtain CMPs.

### Drug Release Performance Test of Drug-Encapsulated CMPs

FITC-BSA and Rhodamine B-labeled eumenitin were used to study the release profile separately. We used a near-infrared light to illuminate CMPs periodically, and a parallel group was set without irradiation for comparison. A fluorescence microscope (OLYMPUS) was used to capture the fluorescence images of the CMPs, and the fluorescence intensity was subsequently processed.

### Hemolysis Test of CMPs

Centrifuged red blood cells were diluted to a final concentration of 5% (v/v) using PBS. HAMA/SFMA inverse opal hydrogel (G III), bulk DEX-BA/DEX-CA dynamic hydrogel (G IV), or CMPs (G V) were mixed with red blood cell solution, respectively, and positive control group (G I: water group) and negative control group (G II: PBS group) were set up. The mixture samples were placed in a constant temperature shaker at 37 °C for 2 h. The absorbance of each centrifugal supernatant was read at 576 nm by using a microplate reader. The same procedure was used to study the effect of CMPs and NIR on hemolysis.

### Biocompatibility Assay of CMPs

NIH 3T3 cells (8000/well) were cultured in the DMEM overnight, and then, various materials including IOMPs and DEX-CA/BA were co-incubated with the cells. Then, CCK-8 test was performed after incubation for 1, 2, and 3 days. For the Live/Dead assay, calcein (AM) and propidium iodide (PI) were used to stain cells at different days. The images were recorded through an inverted fluorescent microscope (ZEISS, Axio Vert.A1) at the last day. The same procedure was used to study the effect of CMPs and NIR on biocompatibility.

### In Vitro Tube Formation Test

Tube formation test was performed by human umbilical vein endothelial cells (HUVECs). Four groups were set, including control group (Ctrl), cells cultured with the leaching solution of CMPs without VEGF (G I), VEGF-loaded CMPs (G II), and VEGF-loaded CMPs with NIR irradiation (G III). For each hole of 48-well plate, 3 × 10^4^ HUVECs were applied. After treated for 4 h, the cells were stained with Calcein-AM and images were taken using a fluorescence microscope.

### In Vitro Antibacterial Experiments

*Escherichia coli* (*E. coli*) and *Staphylococcus aureus* (*S. aureus*) were selected as invasive strains. In brief, the bacterial solution was re-suspended by PBS to a certain concentration based on McFarland standards. Then, four groups were set, including control group (Ctrl), bacteria treated with CMPs without eumenitin (G I), bacteria treated with eumenitin-loaded CMPs (G II), and bacteria treated with eumenitin-loaded CMPs and NIR irradiation (G III).

### In Vivo Wound Healing Assay

Sprague Dawley (SD) (male, 6–7 weeks, 200 g each) were bought from Zhejiang Experimental Animal Center. Animal experiments were performed according to the Laboratory Animal Care and Use Guidelines and were approved by Ethical Committee of Wenzhou Institute, University of Chinese Academy of Sciences (WIUCAS22083102). To establish the diabetic rat model, SD rats were injected with STZ intraperitoneally (55 mg kg^−1^) until the blood glucose value was more than 16 mmol L^−1^. After a diabetic rat model had been successfully constructed, SD rats were randomly divided into six groups and a circular skin incision with the diameter of 1.5 cm was created on the rats’ backs (under pain alleviation) with the introduction of bacterial suspension. Then, the rats were subjected to different interventions, including CMPs (G I), eumenitin-loaded CMPs (G II), VEGF-loaded CMPs (G III), CMPs loaded with both eumenitin and VEGF (G IV), and CMPs loaded with eumenitin and VEGF and subjected to regular NIR irradiation (G V). The wound only received a PBS rinse was denoted as a blank control. For the G V, a NIR light source (808 nm, 1.5 W) was used and the wound was irradiated once a day. The photographs of skin wounds were taken at day 0, 3, 6, 9, and 12, and the skin wound areas were measured. Bacteria were isolated from infected wounds two days after treatment and cultured in LB broth and further coated at agar plate. At day 12, the rats were euthanized, the wounds were snipped and collected, and they were then immersed in 4% paraformaldehyde. Subsequently, the skin wound samples were stained with the H&E assay, Masson staining, immunofluorescence reagents, and immunohistochemical reagents. To assess the in vivo biocompatibility, CMPs were injected subcutaneously into healthy SD rates. At day 10, the rats were euthanized. Then, H&E staining of organs, blood routine and biochemistry were performed to further evaluate.

#### Statistical Analyses

In our experimental results, data are presented as mean ± standard deviation (SD). Student’s *t* test was used for data analysis, and statistical significance levels were denoted as follows: *p < 0.05, **p < 0.01, and ***p < 0.001.

## Results and Discussion

### Fabrication and Characterization of IOMPs

**Fig. 2:**
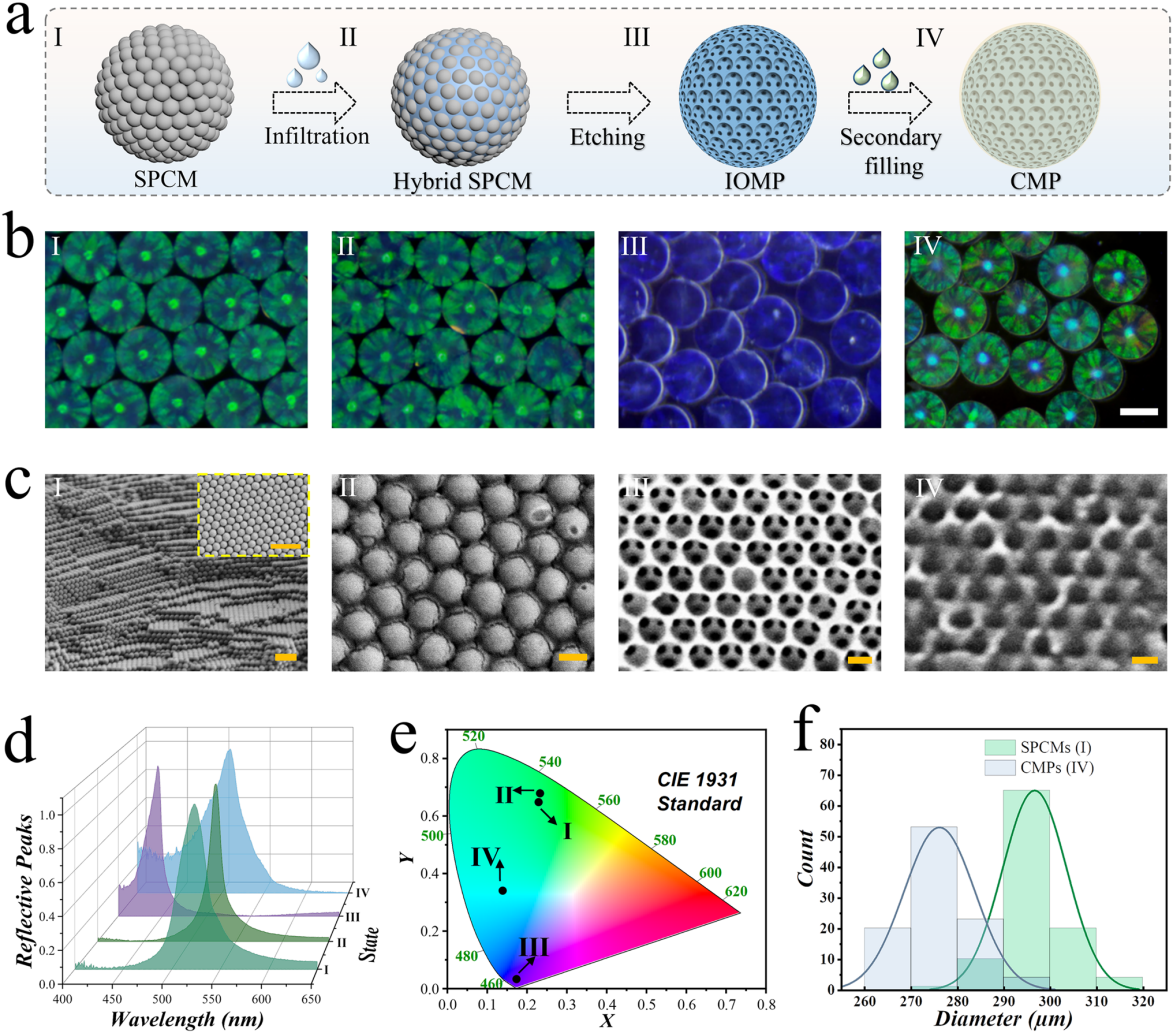
Fabrication and characterization of diverse microspheres. **a** Schematic of the fabrication of the CMP. **b** Microscope images of the relevant hydrogel microspheres corresponding to each stage of the preparation process. **c** SEM images showing the structures of the relevant microspheres. **d** Reflective spectrum and **e** CIE 1931 standard chromaticity diagram of relevant microspheres. **f** Particle size distribution of SPCMs and CMPs. Scale bars are 200 µm in (**b**), 1 µm in (**c**) I, and 200 nm in (**c**) II–IV

In a typical experiment, SPCMs were formed by self-assembly of nanoparticles with the process of gradual dehydration of emulsion droplets. Then, the SPCMs served as sacrificial templates to fabricate inverse opal microsphere scaffolds. Briefly, a pregel solution composed of 10% (w/v) HAMA, 4% (w/v) SFMA, and 1% HMPP effectively filled the interstitial spaces among the silica nanoparticles. Following curing of the pregel solution with an ultraviolet (UV) light source, the hybrid microspheres were separated from the hydrogel block. Subsequently, the silica nanoparticles were etched to obtain the inverse opal hydrogel microsphere scaffolds. The secondary infusion hydrogel was introduced into the IOMP pores through centrifugation or negative pressure, thus achieving CMPs. The production process and characterization of the microspheres at various stages are illustrated in Fig. 2a, b. Notably, SPCM displayed vibrant structural colors under illumination, and this distinctive feature was preserved throughout the fabrication process. Scanning electron microscopy (SEM) was employed to investigate the microscale features of these microspheres. It was observed that silica nanoparticles self-assembled into a hexagonal close-packed structure in the SPCMs (Fig. 2c-I). At the second stage, the interstitial spaces between silica nanoparticles were effectively filled by hydrogel (Fig. 2c-II). Upon removal of the silica, the resulting interconnected porous structure maintained its orderly arrangement (Fig. 2c-III), making it possible for subsequent filling of the secondary hydrogel (Fig. 2c-IV).

Owing to the regularly repeating nanounits, SPCMs, hybrid SPCMs, IOMPs, and CMPs were all endowed with the photonic bandgap (PBG) effect, exhibiting vivid structural color characteristics. The PBG effect is a manifestation of periodic nanounits preventing the propagation of specific light frequencies. This results in the exhibition of vibrant structural colors and the corresponding appearance of reflection peaks, in accordance with Bragg’s formula:

1 $$\lambda = 1.633dn_{{{\text{average}}}}$$

In this equation, *λ* represents the wavelength of the characteristic reflection peak of microspheres, *d* is the distance between adjacent repeating units, and *n*_average_ is the average refractive index of the microsphere system. In this study, the size of the silica nanoparticles used was approximately 240 nm, leading to SPCMs displaying green structural color. The reflection spectrum of the four types of microspheres and their 1931 standard chromaticity diagram are shown in Fig. 2d, e. The characteristic ordered nanostructure and structural color made them suitable for drug delivery and sensor applications. We also analyzed the size distribution of SPCMs and CMPs. As shown in Fig. 2f, both SPCMs and CMPs exhibited size uniformity, and the size of CMPs was slightly smaller compared to the SPCMs.

### Fabrication and Characterization of DEX-CA/BA

**Fig. 3:**
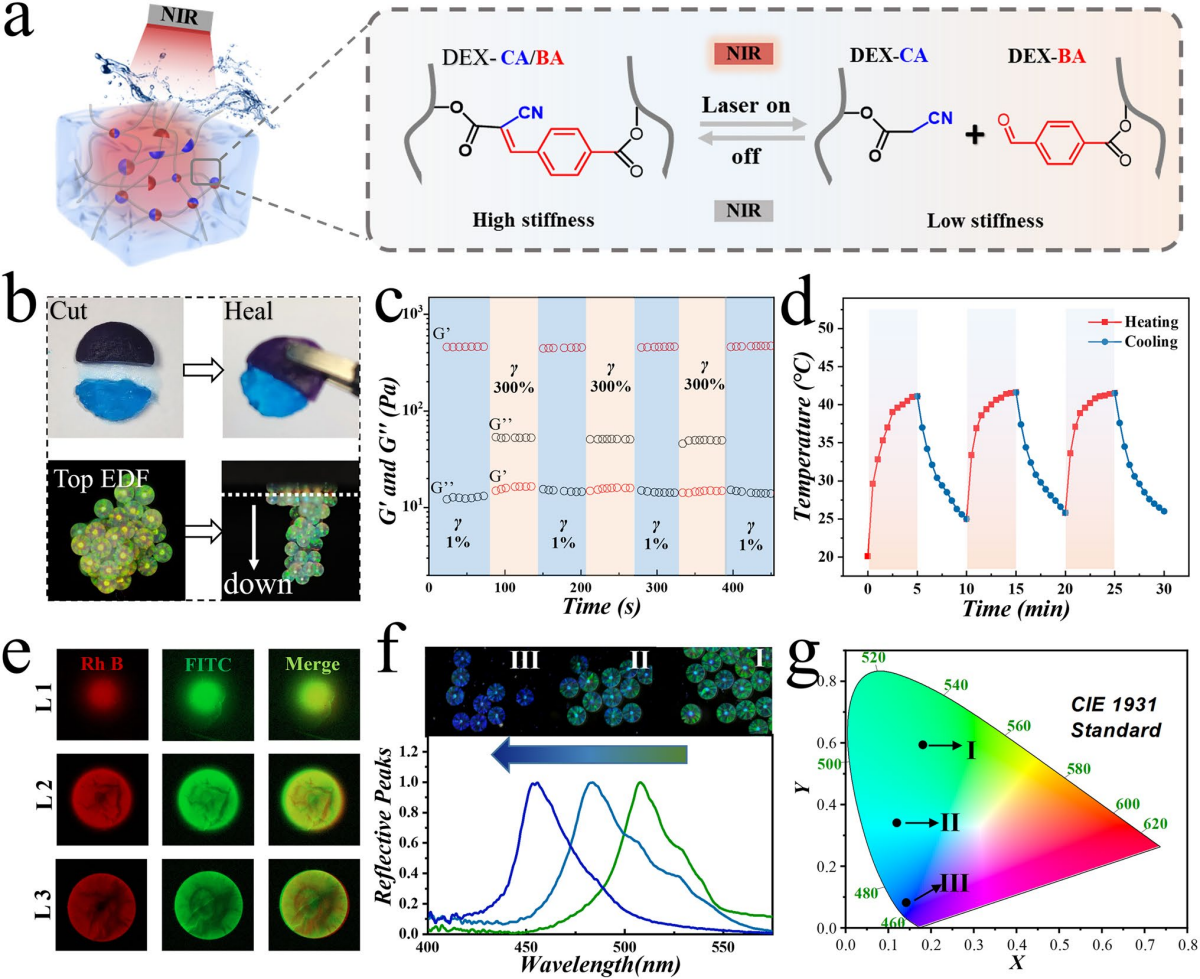
Assessment of the self-healing, NIR responsiveness, and drug release behaviors of CMPs. **a** Schematic of the mechanism of dynamic hydrogel with reversible stiffness changes. **b** Self-bonding/adhesion ability of a bulk dynamic hydrogel (top) and CMPs (bottom). The top view of CMPs was shot through extended depth of focus (EDF). **c** Step strain measurement under alternate strain of 1% and 300% of a bulk DEX-BA/DEX-CA dynamic hydrogel. **d** Cyclic temperature curve of photothermal CMPs. **e** Confocal laser scanning fluorescence images of CMP encapsulated with Rhodamine B-labeled eumenitin and FITC-BSA (Red: Rhodamine B; Green: FITC). **f** Reflective spectrum and **g** CIE 1931 standard chromaticity diagram of the CMPs during the drug release process

In detail, the secondary infusion hydrogel was a dynamic hydrogel composed with DEX-CA and DEX-BA, whose chemical structures are shown in Figs. S1 and S2. As described in our previous study, the dynamic hydrogel was formed through the KC reaction, and the presence of histidine can promote the reaction (Fig. S3). The characterized peak of C=C was found in FT-IR spectra of dynamic hydrogel, indicating the connection between DEX-CA and DEX-BA (Fig. S4). In addition, the thermal response of the DEX-CA/BA hydrogel triggered by histidine was also studied. It was observed that the 6% (w/v) hydrogel remained at high stiffness at 37 °C and switched to a low stiffness at 42 °C (Fig. S5). This situation is conducive to in vivo drug delivery. Thus, the hydrogel with the concentration of 6% was chosen for the subsequent experiments. To vividly study the gelation of DEX-CA/BA, SEM and rheometer were utilized to characterize the morphology and rheology property (Figs. S6 and S7). When infused in IOMPs, the photothermal responsiveness of BPQDs-loaded CMPs helped DEX-CA/BA hydrogel achieve reversible switching between high and low stiffness upon NIR irradiation, as illustrated in Fig. 3a. On the other hand, it was noteworthy that the dynamic chemical bonds contributed to the exceptional self-healing properties of DEX-CA/BA hydrogels. As a result, CMPs also acquired a degree of self-healing capability (Fig. 3b). Alternate step–strain sweep measurement indicated that the dynamic infusion hydrogel experienced structure breakdown under high strain conditions but swiftly recovered when the strain returned to 1% (Fig. 3c).

The power of NIR irradiation and the concentration of BPQDs were crucial factors affecting the heating process and platform temperature (Fig. S8). To meet the requirements for controllable drug release and dynamic switching of hydrogel stiffness, a concentration of 0.2 mg mL^−1^ of BPQDs and an irradiation power of 1.5 W were selected. Through repeated photothermal control cycles, it was demonstrated that the constructed BP-loaded IOMPs exhibited stable and outstanding photothermal performance (Fig. 3d). Based on these features, the CMPs exhibited the potential to serve as excellent carriers for drug release. Considering that the properties of hydrogels may be affected by various ingredients, the effects of drugs on the gelation process and modulus of dynamic hydrogels are studied. It was found that experimental concentrations of the drug have little effect on dynamic hydrogel (Fig. S9). To simulate drug loading and release, Rhodamine B-labeled eumenitin and FITC-BSA were used. These two fluorescent substances were encapsulated within DEX-CA/BA hydrogel precursor and subsequently filled into IOMPs. Fluorescence images obtained through confocal microscopy revealed uniform drug loading within the CMPs (Fig. 3e). During the simulated drug release process, it was evident that NIR irradiation can effectively control drug release (Fig. S10). This could be attributed to the decrease in stiffness of the DEX-CA/BA hydrogel under photothermal conditions. Meanwhile, the partial release of filling hydrogel and encapsulated-drug under stimulation of NIR irradiation would induce the changes in average refractive index of the CMPs which also changed correspondingly. According to the Bragg’s formula, this change will lead to the change of the visual structure color of the CMPs. In this process, the CMPs and the corresponding reflection peak, as well as the 1931 standard chromaticity diagram, are shown in Fig. 3f, g.

### In Vitro Characterization of CMPs

**Fig. 4:**
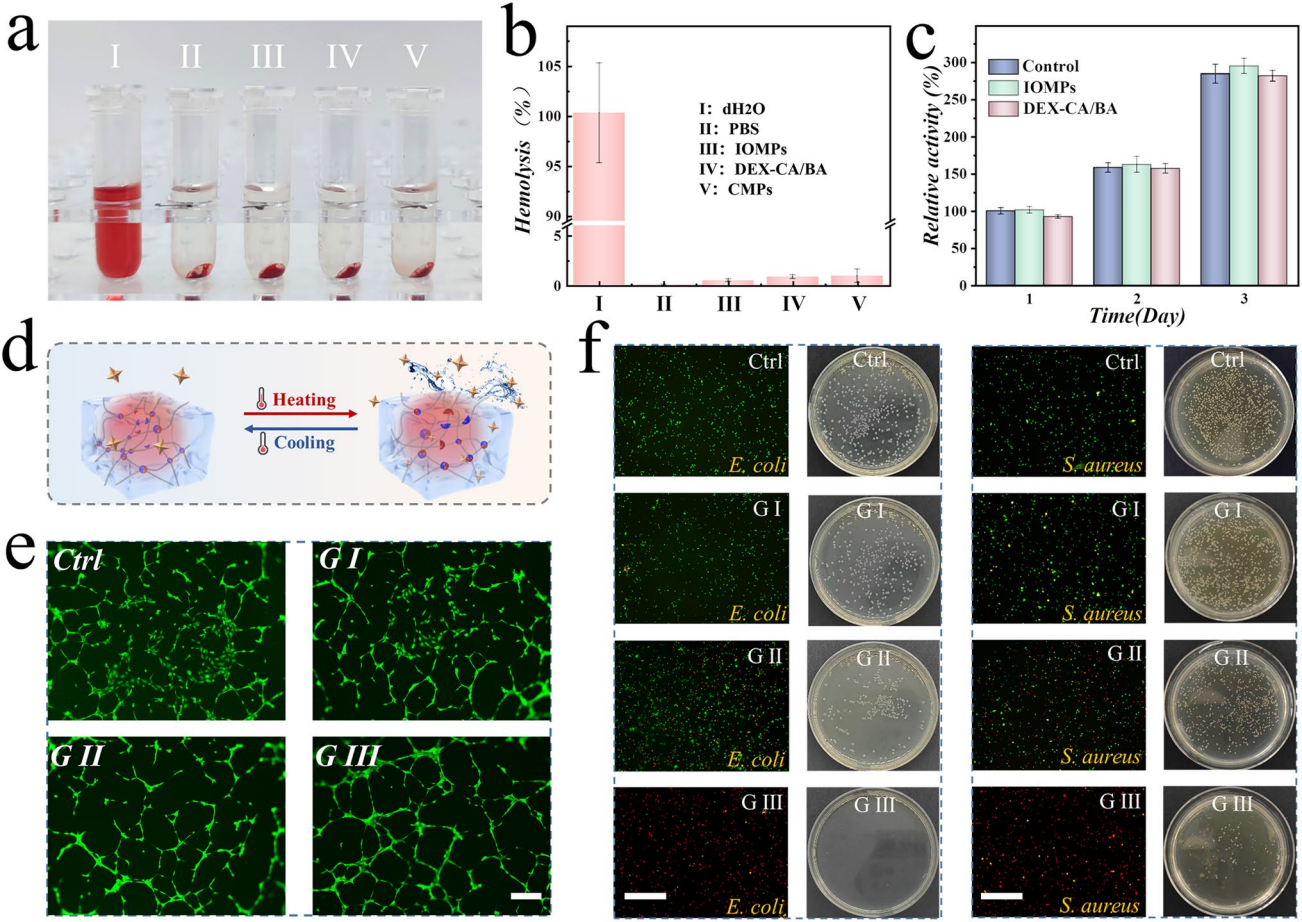
Properties of angiogenesis, antibacterial activity, hemo-compatibility, and biocompatibility of the CMPs. **a, b** Images and the estimated hemolysis rate of different materials including IOMPs, DEX-CA/BA, and CMPs. **c** Statistical analysis of biocompatibility of IOMPs and DEX-CA/BA. **d** Schematic of NIR irradiation promoting drug release from dynamic hydrogel. **e** Typical images of tube formation of HUVECs in different groups including control group, CMPs without VEGF (G I), CMPs with VEGF (G II), and CMPs with VEGF and NIR irradiation (G III), respectively. **f** Live/dead fluorescent images and corresponding plate coating photographs with different treatments, including control group, CMPs without eumenitin (G I), CMPs with eumenitin (G II), and CMPs with eumenitin plus NIR irradiation (G III), respectively. Scale bars are 200 µm in (**e**) and 150 µm in (**f**)

As the CMPs are aimed to be applied in tissue repair, it is imperative to investigate their hemo-compatibility and biocompatibility. As depicted in Figs. 4a, b and S11, both IOMPs and bulk DEX-CA/BA hydrogel, as well as CMPs, all exhibited the low hemolysis rates (< 5%), indicating the satisfactory hemo-compatibility. Furthermore, when co-cultured with 3T3 cells, the experimental groups treated with IOMPs, DEX-CA/BA, as well as CMPs plus NIR irradiation, possessed good cell viability and the live/dead assay results, which were similar to those of the control group (Figs. 4c and S12, S13), indicating that the CMPs system was non-cytotoxic. Meanwhile, the attachment and proliferation of NIH 3T3 cells on CMPs were also noticed (Fig. S14). These results suggested that the proposed CMPs exhibited outstanding biocompatibility.

Moreover, we separately validated the functions of VEGF and eumenitin-loaded CMPs in promoting angiogenesis and fighting against bacteria. In view of the NIR-stimulated release capacity of CMPs (Fig. 4d), we compared the effects with and without NIR irradiation. Human umbilical vein endothelial cells (HUVECs) were subjected to various treatments, including the Control group, G I (CMPs), G II (VEGF-loaded CMPs), and G III (VEGF-loaded CMPs plus NIR irradiation). The results demonstrated that cells in G III exhibited superior angiogenic effects compared to the other groups (Figs. 4e and S15). Bacterial infections remain important causes for the treatment of wounds. The antibacterial capacity of eumenitin-loaded CMPs was evaluated against *E. coli* and *S. aureus*. Figure 4f showed that the majority of bacterial were killed in G III which were treated with eumenitin-loaded CMPs under NIR irradiation. As a comparison, almost no dead bacteria were observed for the G II group without using NIR irradiation, which was comparable to the control group. Besides, the bacteria treated with NIR irradiation only did not exhibited the same bacteria-killing ability as GIII. Hence, the better antibacterial property of G III could be attributed to a higher release of the antibacterial drug from eumenitin-loaded CMPs under NIR irradiation. Furthermore, the agar plate assay also indicated that the G III group showed obvious inhibition influence on the bacteria growth when compared to G III group. Taken together, CMPs could be severed as intelligent and controllable drug carriers for further applications combined with NIR irradiation.

### In Vivo Wound Healing Assay of CMPs

**Fig. 5:**
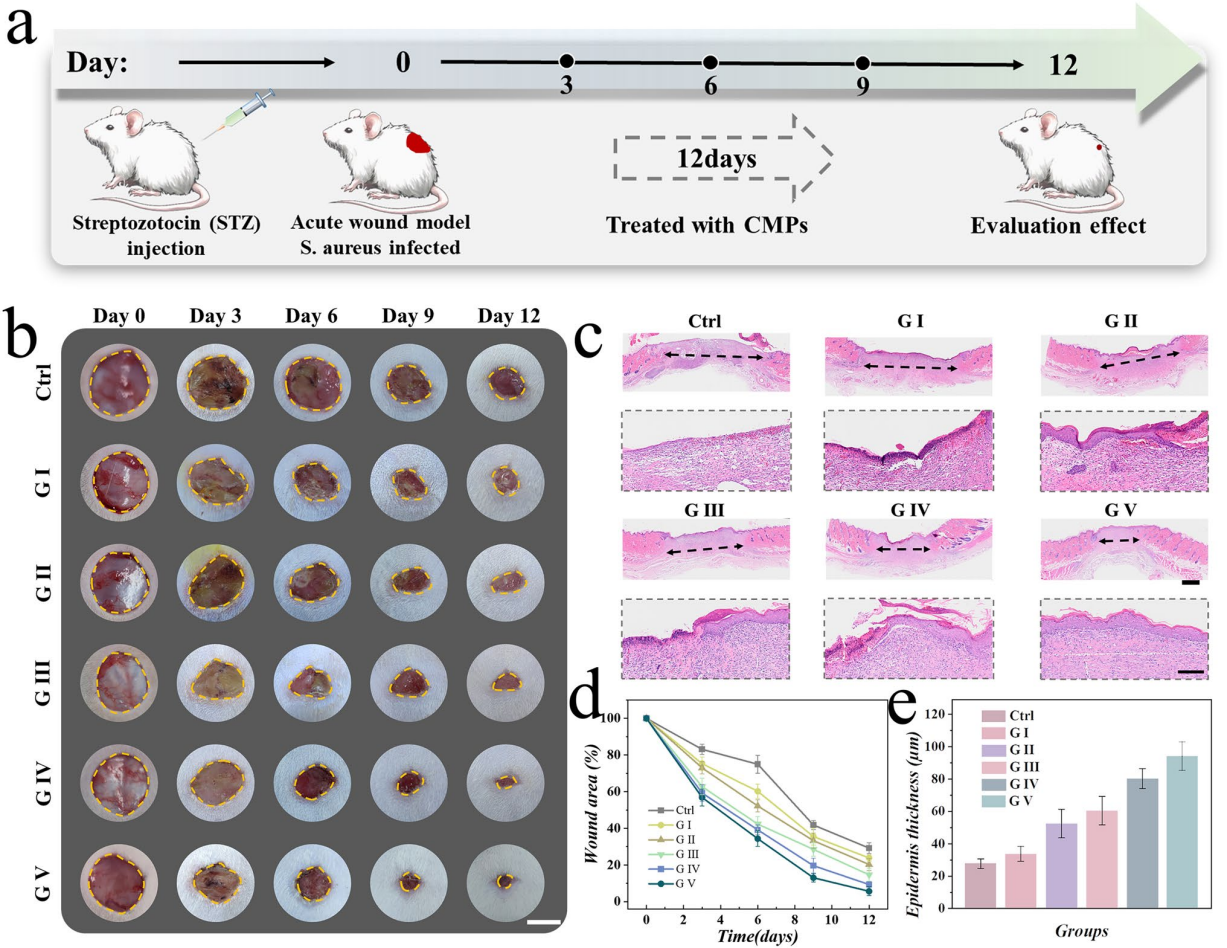
Evaluation of in vivo treatment efficacy on chronic wound healing of CMPs. **a** Schematic of the animal experimental schedule. **b** Optical images recorded of each group of mice wounds every three days. **c** Corresponding H&E staining images and epidermal details of each group’s wound in Day 12. **d** Data analysis of wound area in different groups on diverse days. **e** Data analysis of epidermis thickness in different groups on day 12. Scale bars are 10 mm in (**b**), 1 mm in (**c**) (top), and 200 µm in (**c**) (bottom)

The in vivo wound healing efficacy of CMPs was evaluated in a full-thickness chronic, diabetic infected wound model. The SD rat diabetes model was established through intraperitoneal injection of streptozotocin (STZ). After maintaining blood glucose at a certain concentration, circular full-thickness skin wounds were created on the rats’ backs (Fig. 5a). Subsequently, rats in different groups were subjected to various interventions to assess the effectiveness of wound healing promotion of CMPs. These interventions included a Control group (PBS), G I (CMPs without drugs), G II (CMPs loaded with eumenitin), G III (CMPs loaded with VEGF), G IV (CMPs loaded with eumenitin and VEGF), and G V (CMPs loaded with eumenitin and VEGF, plus NIR irradiation). CMPs were applied to the wound with the help of an injection needle, and their structural color could also been observed (Fig. S17). As illustrated in Fig. 5b, wound images were regularly recorded. Bacteria were isolated from infected wounds two days after treatment and cultured in LB broth and further coated at agar plate. As shown in Fig. S18, after treated with CMPs plus NIR irradiation, a small amount of bacteria showed up on the infected wound, indicating that CMPs still had obvious antibacterial activity in vivo*.* The rats were euthanized on day 12, and skin samples were then collected from the wound sites for evaluation. Due to the prolonged hyperglycemic pathological microenvironment, the closure rate of wounds in the Control group was significantly lower than in other groups. In contrast, the skin in G V exhibited the most optimal wound closure effect. Furthermore, the pathological features of the newly formed tissues were assessed through hematoxylin and eosin (H&E) staining (Fig. 5c). Group V exhibited the narrowest wound width and a relatively more complete degree of epithelialization. After performing a statistical analysis of the wound closure area and regenerated epithelial thickness, the group treated with CMPs system loaded with dual drugs, in conjunction with NIR irradiation, demonstrated superior wound healing effects compared to other groups (Fig. 5d, e). These suggested that the NIR-controlled irradiation and the intelligent responsiveness of CMPs together played a significant role in promoting wound healing.

### Evaluation of Pathological Features on Wounds

**Fig. 6:**
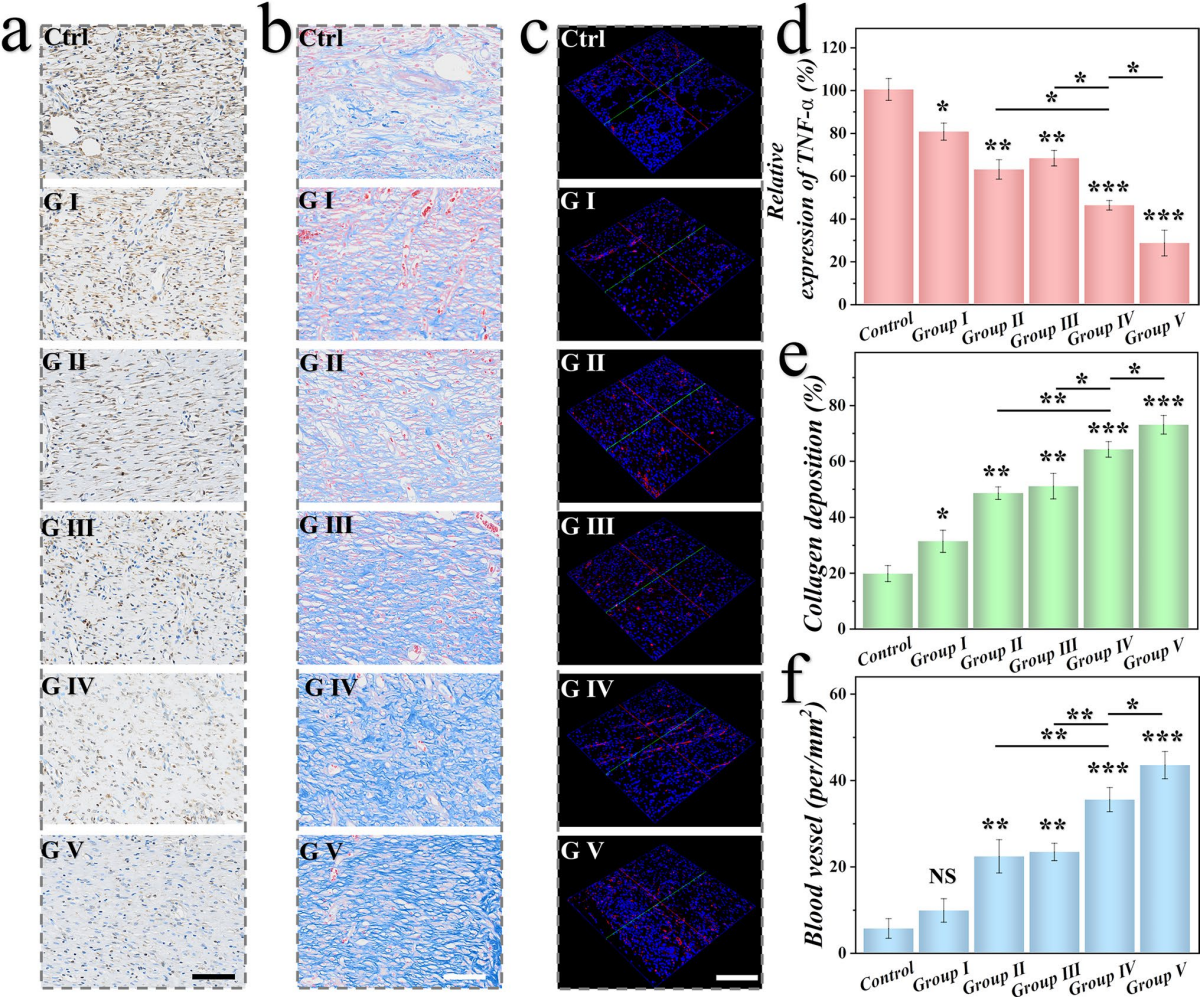
Evaluation of pathological features on chronic wounds in different groups. **a** Immunohistochemical staining of TNF-α in different groups. **b** Staining of collagen deposition in different groups. **c** Immunofluorescent staining of CD31 in different groups. Data analysis of **d** relative expression of TNF-α, **e** collagen deposition, and **f** blood vessel structures in each group. Scale bars are 100 µm in (**a**) and (**b**), 200 µm in (**c**)

Bacterial infection is the predominant cause that delayed wound healing of chronic wounds. To further evaluate the expression of inflammatory factors in the wound tissue of each group, we performed immunohistochemical staining of tumor necrosis factor-α (TNF-α) on the wound tissue. As shown in Fig. 6a, d, it was seen that the expression of TNF-α was higher in the control group and G I than the other groups, suggesting that the wounds remained in inflammatory stage. Additionally, Masson staining was conducted to assess collagen deposition at the wound site. As depicted in Fig. 6b, the alignment and density of collagen protein were more pronounced in the drug-loaded CMPs groups, and further there was more collagen formed, confirming the CMPs’ ability to promote extracellular matrix deposition. Furthermore, neoangiogenesis plays a crucial role in wound repair, and to investigate the impact of VEGF on wound healing, we used immunofluorescence staining of CD31 to characterize the newly formed blood vessels. Fluorescent images in Fig. 6c revealed minimal CD31-positive areas in the control group, while the CMPs@AMP&VEGF + NIR group exhibited the highest CD31 expression. Moreover, quantitative analysis of TNF-α, collagen deposition, and blood vessel density indicated that the CMPs can promote collagen deposition and stimulate angiogenesis (Fig. 6d-f). To further assess the in vivo biocompatibility, CMPs were injected subcutaneously into healthy rates. At day 10, rats were euthanized. It was indicated that no obvious pathological abnormality or elevation found in H&E staining of main organs and blood routine and biochemistry (Figs. S19 and S20). Therefore, in combination with its structural color sensing features, the CMPs represent a highly promising dressing material for wound healing management.

## Conclusion

In summary, we propose innovative composite microspheres (CMPs) based on structural color hydrogel microspheres for wound management. The CMP comprised a photothermal-responsive inverse opal framework constructed by HAMA and SFMA doped with BPQDs and was further re-filled by a KC reaction-based dynamic hydrogel containing eumenitin and VEGF. Notably, the CMPs can be applied arbitrarily, and they can adhere together upon NIR irradiation by leveraging the BPQDs-mediated photothermal effect and the thermoreversible stiffness change of the dynamic hydrogel. Additionally, the release behavior of eumenitin and VEGF can be regulated by the same mechanism. Moreover, effective monitoring of the drug release process can be achieved through visual color variations. The CMPs system has demonstrated desired capabilities of controllable drug release and efficient wound management. These characteristics suggest great value of the proposed CMPs as wound dressings for related biomedical applications.

## Supplementary Information

Below is the link to the electronic supplementary material.

Supplementary file1 (PDF 1218 KB)
