# Supplementary material for: Self-Healing Dynamic Hydrogel Microparticles with Structural Color for Wound Management
Source: Nanomicro Lett. 2024 Jul 2;16:232. doi: 10.1007/s40820-024-01422-4 (PMC11219637; doi:10.1007/s40820-024-01422-4)
Supplement: Supplementary file 1 — Supplementary file1 (PDF 1218 KB) [file 40820_2024_1422_MOESM1_ESM.pdf]

Supporting Information for

Self-Healing Dynamic Hydrogel Microparticles with Structural Color for Wound Management

Li Wang[1,2,3], Xiaoya Ding[4], Lu Fan[3,4], Anne M. Filppula[3], Qinyu Li[1,*], Hongbo Zhang[1,3,*], Yuanjin Zhao[1,2,*], Luoran Shang[1,4,5,*],

[1] Department of General Surgery, Ruijin Hospital, Shanghai Jiaotong University School of Medicine, Shanghai 200025, P. R. China

[2] Department of Rheumatology and Immunology, Nanjing Drum Tower Hospital, School of Biological Science and Medical Engineering, Southeast University, Nanjing 210096, P. R. China

[3] Pharmaceutical Sciences Laboratory, Åbo Akademi University, Turku 20520, Finland

[4] Oujiang Laboratory (Zhejiang Lab for Regenerative Medicine, Vision and Brain Health), Wenzhou Institute, University of Chinese Academy of Sciences, Wenzhou 325001, P. R. China

[5] Shanghai Xuhui Central Hospital, Zhongshan-Xuhui Hospital, and the Shanghai Key Laboratory of Medical Epigenetics, the International Co-laboratory of Medical Epigenetics and Metabolism (Ministry of Science and Technology), Institutes of Biomedical Sciences, Fudan University, Shanghai, 200032, P. R. China

*Corresponding authors. E-mail: lqy10807@rjh.com.cn (Qinyu Li); hongbo.zhang@abo.fi (Hongbo Zhang); yjzhao@seu.edu.cn (Yuanjin Zhao); luoranshang@fudan.edu.cn (Luoran Shang)

Supplementary Figures

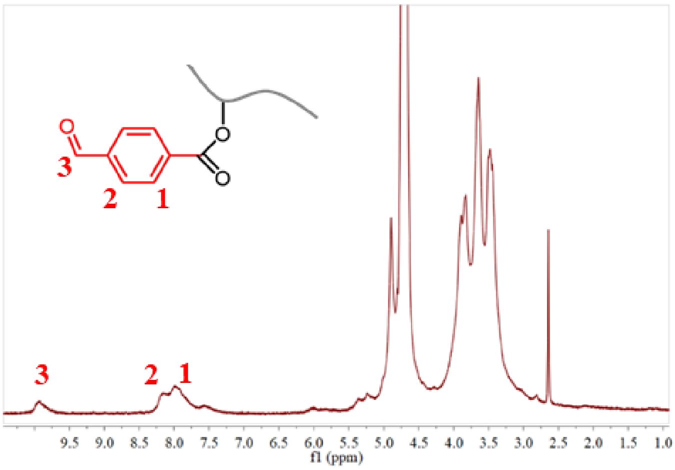

Fig. S1 H-NMR spectrum of DEX-BA

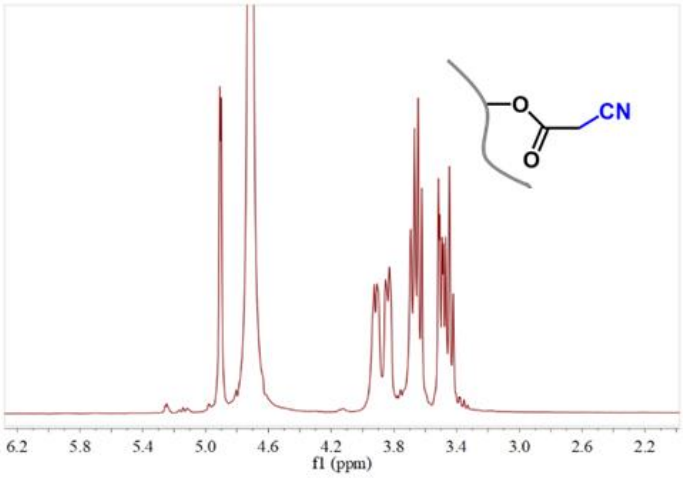

Fig. S2 H-NMR spectrum of DEX-CA

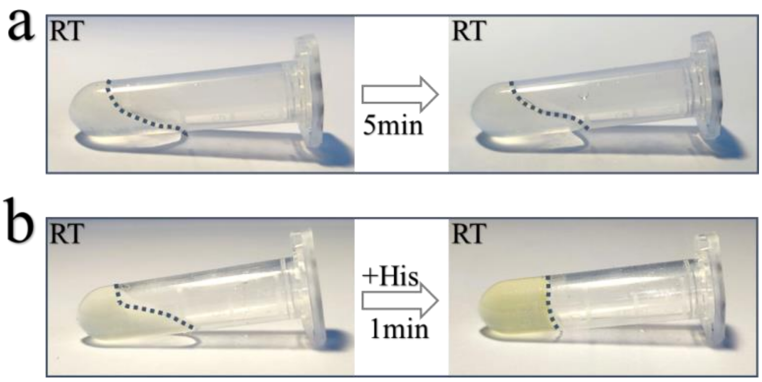

Fig. S3 Images showing that histidine promotes gelation of the dynamic hydrogel. When the pictures were taken, the tube was changed from the upright state to the inclined state. The dashed lines represent the outline of the sample

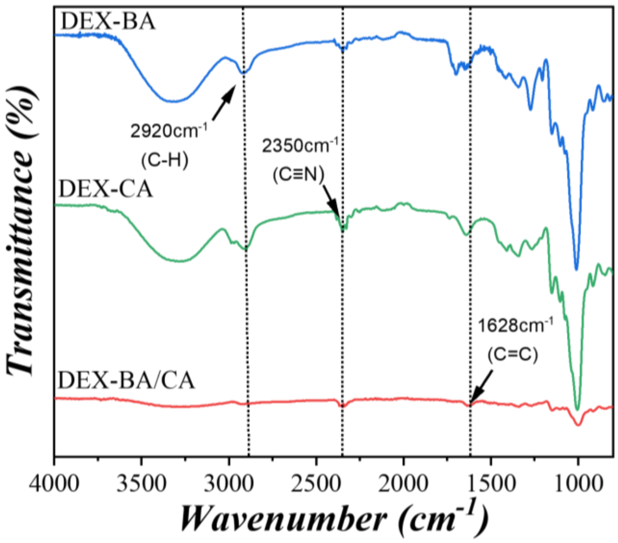

Fig. S4 FT IR spectra of DEX-BA, DEX-CA and dynamic hydrogel

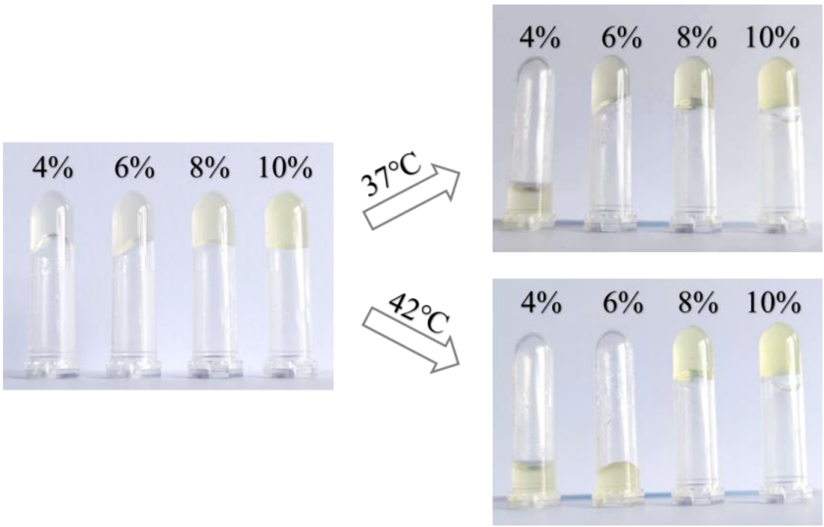

Fig. S5 Temperature sensitivity of dynamic hydrogels with different concentrations

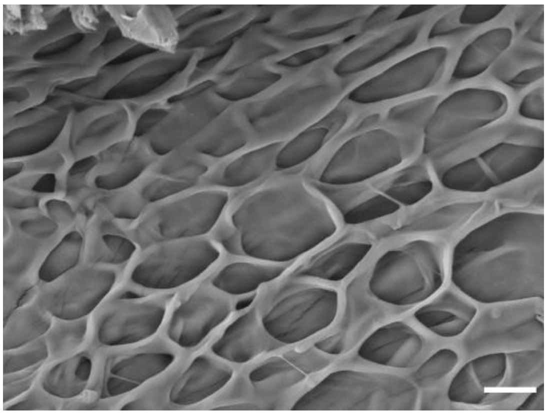

Fig. S6 SEM image of DEX-CA/BA dynamic hydrogel. Scale bar is 10 μm

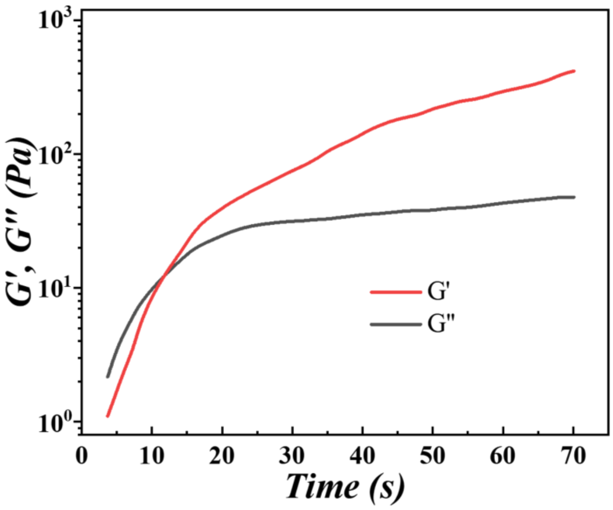

Fig. S7 Real-time rheology test of DEX-CA/BA dynamic hydrogel

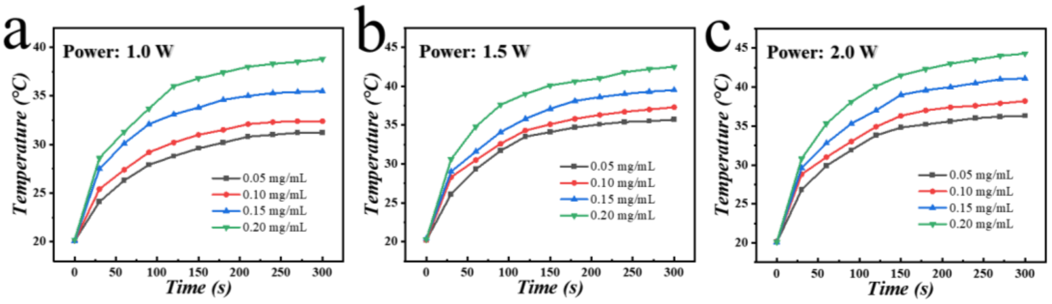

Fig. S8 Temperature rising curve of the photothermal hydrogel under different NIR power irradiation

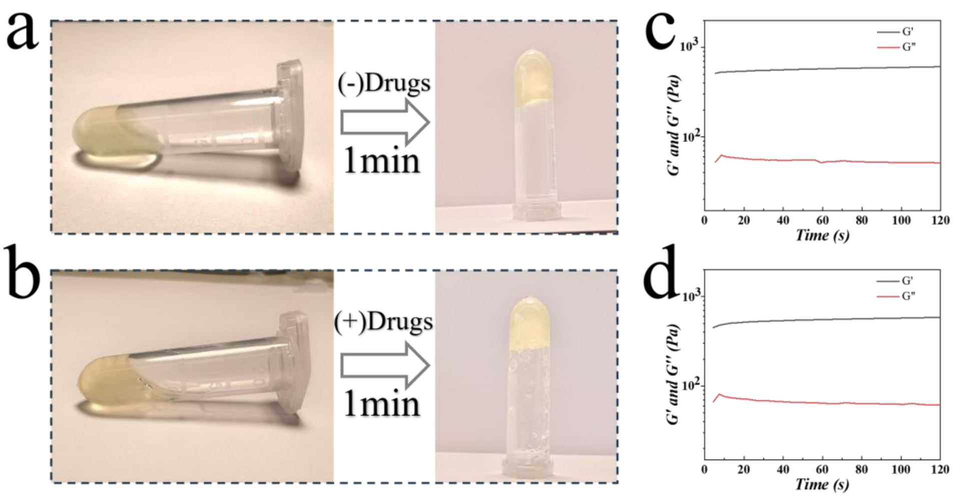

Fig. S9 a-b Images of gelation process of DEX-CA/BA with or without eumenitin and VEGF. **c** Rheology test of the DEX-CA/BA without drugs. **d** Rheology test of the DEX-CA/BA with drugs

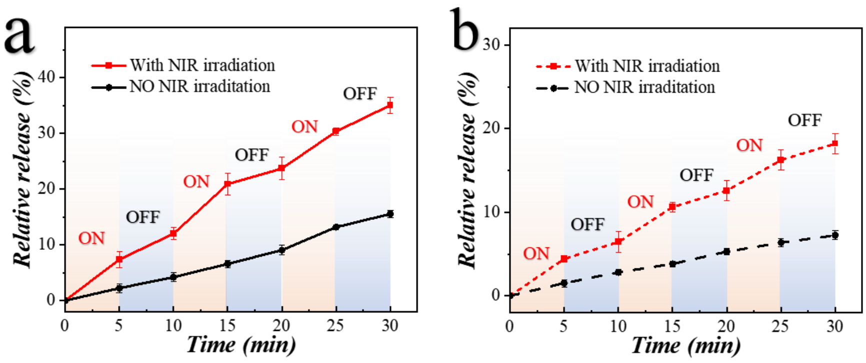

Fig. S10 Drug release curve of Rhodamine B labeled-eumenitin and FITC-BSA with or without NIR irradiation

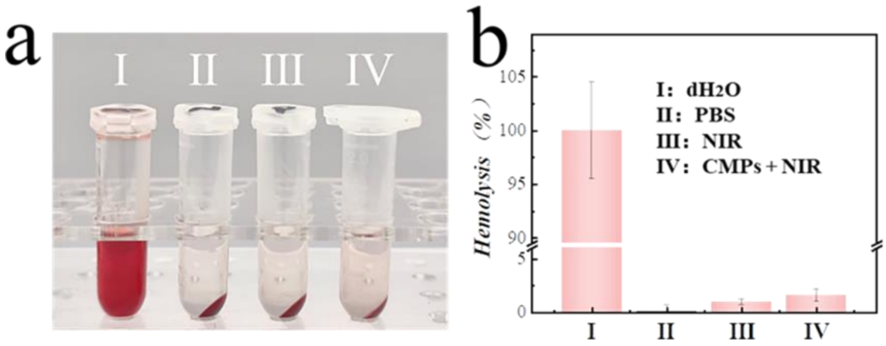

Fig. S11 a-b Images and the estimated hemolysis rate of different treatments including control group, NIR irradiation, and CMPs plus NIR irradiation

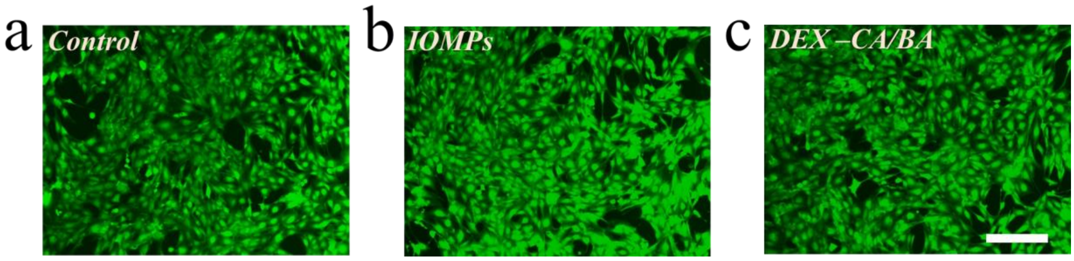

Fig. S12 Fluorescent images of NIH 3T3 cells treated with different strategies. Scale bar represents 200 μm.

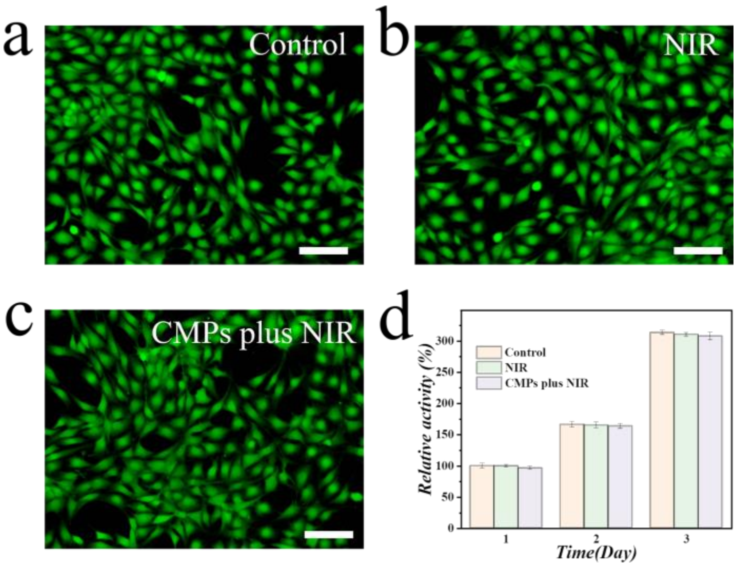

Fig. S13 a-c Fluorescent images of NIH 3T3 cells treated with different strategies including control group, NIR irradiation, and CMPs plus NIR irradiation. **d** Analysis of relative activity of NIR 3T3 cells in different groups. Scale bars are 50 μm

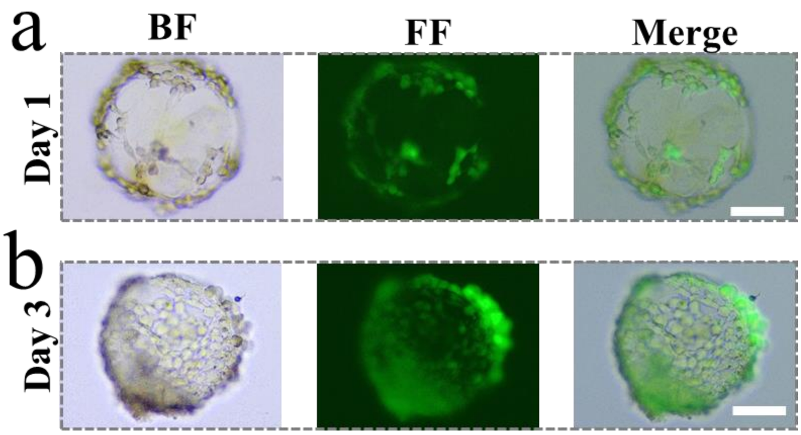

Fig. S14 Bright field and fluorescent images of CMPs co-culturing with NIH 3T3 cells

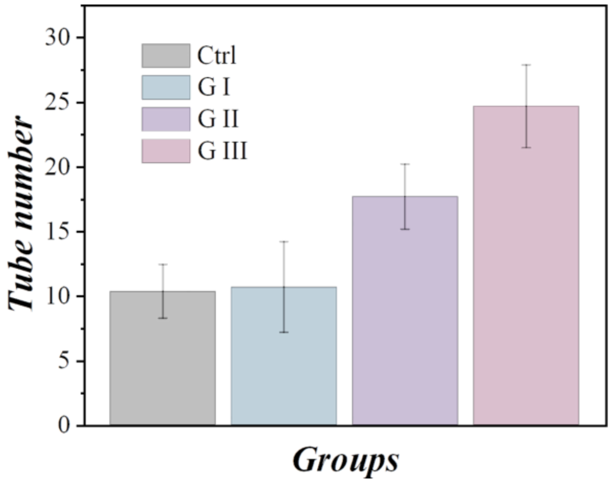

Fig. S15 Data analysis of tuber number in different groups

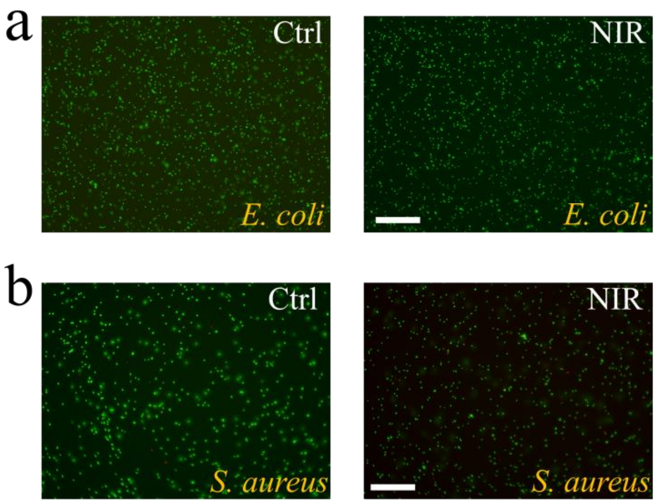

Fig. S16 Live/dead fluorescent images of bacteria with different treatments, including control group and NIR irradiation. Scale bars are 100 μm

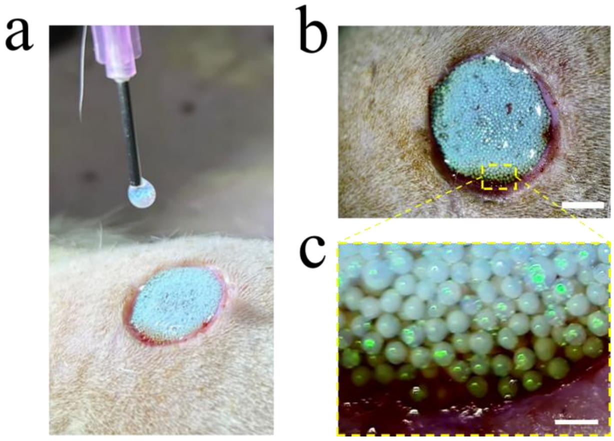

Fig. S17 a CMPs applied on the wound. **b-c** Images of CMPs-treated wound and local magnification of structural color microspheres. Scale bars are 5 mm in (**b**) and 500 μm in (**c**)

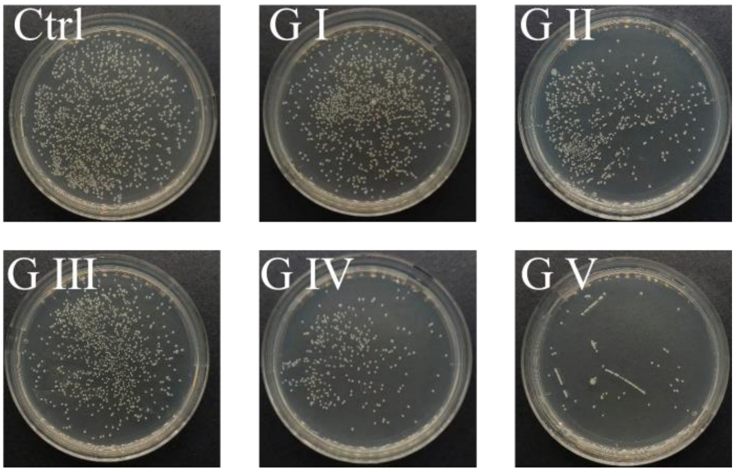

Fig. S18 Representative images of bacterical colonies separated from wounds with different treatments

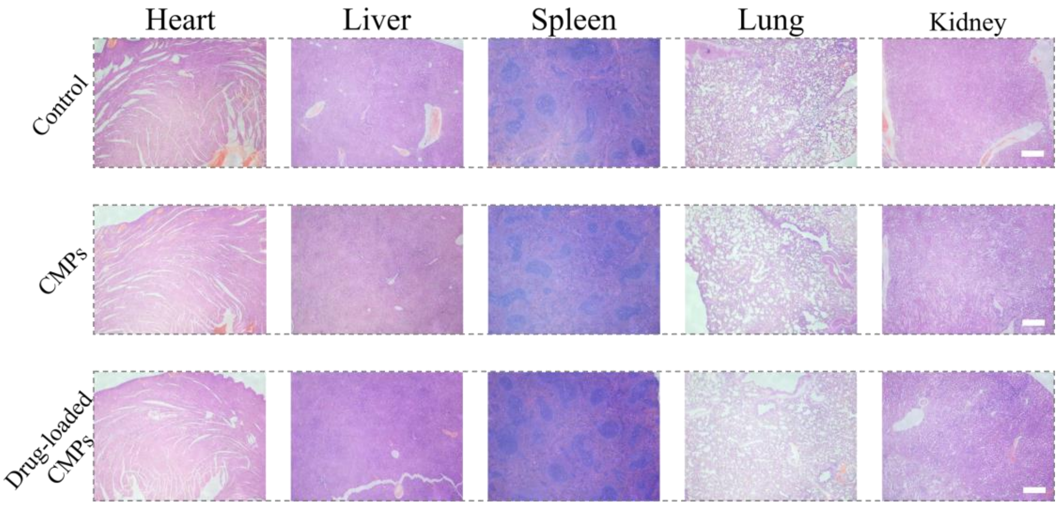

Fig. S19 H&E staining images of main organs from rats in different groups. Scale bars are 500 μm

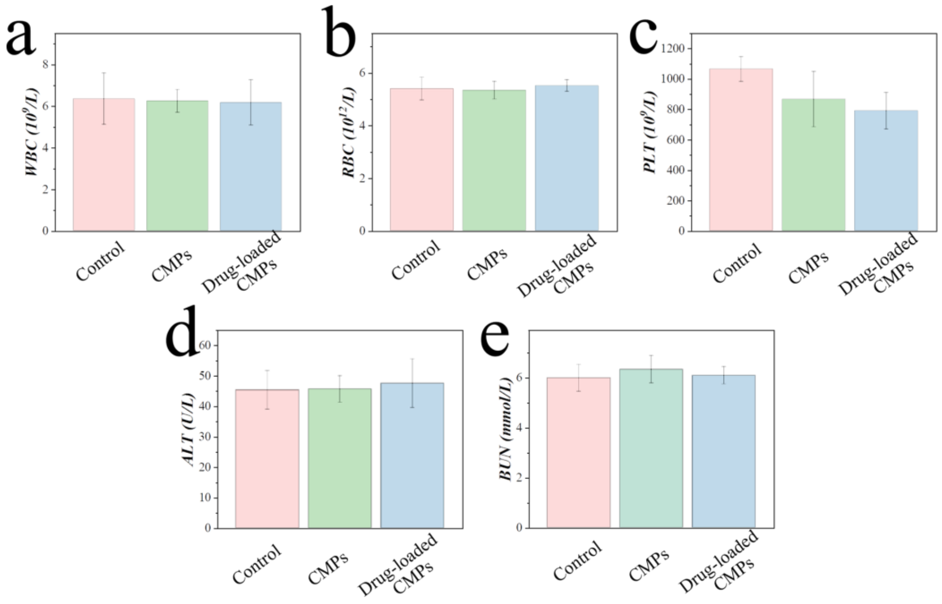

Fig. S20 Blood routine and biochemisity analysis of rats in different groups
